# Establishment and evaluation of a model for clinical feature selection and prediction in gout patients with cardiovascular diseases: a retrospective cohort study

**DOI:** 10.3389/fendo.2025.1599028

**Published:** 2025-10-10

**Authors:** Bingbing Fan, Yuqing Ye, Zihan Wang, Yuanyuan Xu, Meishan Lu, Weihong Cong, Fang Ma

**Affiliations:** ^1^ Xiyuan Hospital, China Academy of Chinese Medical Sciences, Beijing, China; ^2^ Graduate School, Beijing University of Chinese Medicine, Beijing, China; ^3^ Graduate School, Heilongjiang University of Chinese Medicine, Harbin, China

**Keywords:** gout, cardiovascular events, prediction nomogram, machine learning (ML), nomo gram

## Abstract

**Background:**

Gout is a chronic inflammatory condition increasingly recognized as a risk factor for cardiovascular events (CVE). Early identification of high-risk individuals is crucial for targeted prevention and management. However, conventional risk stratification approaches often fall short in accuracy and clinical utility. This study aimed to develop and validate a robust, interpretable machine learning (ML)-based model for predicting CVE in patients with gout.

**Methods:**

This retrospective cohort study included 686 hospitalized gout patients at Xiyuan Hospital (Beijing, China) between January 1, 2013, and December 31, 2023. We applied Synthetic Minority Oversampling Technique (SMOTE) combined with random undersampling of the majority class. Then, patients were randomly divided into training (70%) and testing (30%) sets. A comprehensive set of clinical and biochemical variables (n = 39) was collected. Feature selection was performed using Boruta algorithms and Lasso to identify the most predictive variables. Multiple ML algorithms—including Decision Tree Learner, LightGBM Learner, K Nearest Neighbors Learner, CatBoost Learner, Gradient Boosting Desicion Tree Learner—were implemented to construct predictive models. SHAP values were used to assess model interpretability, and robustness was evaluated through 10-fold bootstrap resampling with enhanced standard error estimation.

**Results:**

Of the 686 patients, 263 experienced cardiovascular events during follow-up (incidence rate: 38.3%). A logistic regression model was constructed based on eight variables selected using the Boruta feature selection algorithm: sex, age, PLT, EOS, LYM, CO2, GLU and APO-B. Among the five models evaluated, the CatBoost classifier achieved the best performance, with the highest area under the ROC curve (AUC) of 0.976 and the recall of 0.971. Furthermore, SHAP (SHapley Additive exPlanations) values were employed to provide both global and individual-level interpretability of the CatBoost model. To assess the model’s generalization performance, bootstrap resampling was performed 10 times. Based on these results, the standard error was improved using machine learning-based enhancement methods, thereby optimizing the model’s robustness and predictive stability.

**Conclusion:**

The logistic regression analysis revealed that age (OR=1.351, p<0.001), CO2 (OR=0.603, p=0.004), eosinophil count (OR=2.128, p=0.001), and platelet count (OR=0.961, p<0.001) were significantly associated with the outcome, indicating their potential roles as independent predictors. Notably, while APO_B (p=0.138) and sex (p=0.132) showed no significant association, glucose levels (OR=2.1, p=0.066) exhibited a marginal trend toward significance, warranting further investigation. This tool may support clinicians in identifying high-risk individuals, enabling early interventions and optimized management strategies.

**Limitations:**

This study has several limitations. First, the analysis was based on a single-center dataset, which may limit the generalizability of the findings. External validation in multi-center and prospective cohorts, along with an expanded sample size, is warranted to confirm these results. Second, key confounding factors such as medication use, lifestyle habits, and gout flare frequency were not included in the analysis; future studies should incorporate these variables to provide a more comprehensive assessment.

## Introduction

Gout, a chronic crystal arthropathy pathologically characterized by monosodium urate (MSU) crystal deposition in synovial fluid and periarticular tissues (1), manifests during acute flares as a classic triad of symptoms: abrupt-onset excruciating pain (visual analog scale [VAS] score ≥7), localized hyperthermia (ΔT≥2.1 °C), and erythematous swelling (≥15% periarticular circumference expansion) (2). Data from the Global Burden of Disease Study reveal a persistent upward trajectory in gout prevalence from 1990 to 2025, with projections indicating that the total global prevalence of gout will escalate to 95.8 million cases by 2050 (3, 4). Notably, East Asians exhibit the highest age-standardized prevalence rates, demonstrating statistically significant elevation compared to high-income Western demographic cohorts (5). The diagnostic threshold for hyperuricemia (HUA) (6), defined by international guidelines as serum uric acid (SUA) concentration ≥420 μmol/L (7 mg/dL), demonstrates a global prevalence of 13.4% (7, 8). This metabolic aberration exhibits dose-response relationships with multiorgan dysfunction: each 60 μmol/L SUA increment corresponds to 47% elevated metabolic syndrome risk. Crucially, HUA-gout-cardiovascular diseases (CVDs) forms a vicious triad through interconnected mechanisms (9). Firstly, direct crystal-mediated vascular injury: MSU crystals within vascular walls activate NLRP3 inflammasomes, inducing 3.8-fold IL-1β hypersecretion and enhancing neutrophil extracellular trap (NET) formation to 71.3% (*vs*. 9.8% in healthy controls). These processes synergistically degrade endothelial glycocalyx (42% thickness reduction) and elevate platelet activation markers (2.8-fold sP-selectin increase). This chronic low-grade inflammation (10, 11), compounded by oxidative stress (157% malondialdehyde elevation), accelerates atherosclerotic plaque progression (12) (annual volume growth rate +18.7%). Furthermore, endothelial dysfunction cascade: Chronic HUA reduces nitric oxide bioavailability by 62% while elevating von Willebrand factor (vWF) levels 2.3-fold, collectively promoting atherosclerotic plaque formation (12) (annual volume growth rate +22.4%). Then, metabolic synergy amplification: Gout patients with comorbid hypertension and diabetes demonstrate 3.1-fold higher cardiovascular mortality risk compared to uncomplicated gout cases (13–15). Although consensus exists regarding elevated CVDs risks in gout (17% increased all-cause mortality, 29% CVDs-specific mortality), current risk prediction tools remain suboptimal. Our study addresses this gap through multidimensional parameter integration, developing a visual nomogram model demonstrating superior predictive accuracy versus conventional scoring systems. This instrument enables rapid high-risk patient identification (risk threshold ≥15%) in outpatient settings, establishing a novel paradigm for precision medicine implementation.

## Methods

### Study design and population

This retrospective cohort study included 686 hospitalized patients diagnosed with gout at Xiyuan Hospital in Beijing, China, between January 1, 2013, and December 31, 2023. The study protocol was approved by the Ethics Committees of Xiyuan Hospital, China Academy of Chinese Medical Sciences (2023XLA026-3) with a waiver for informed consent. Inclusion criteria comprised patients with a confirmed clinical diagnosis of gout and complete hospitalization records. The primary outcome was the occurrence of CVDs during hospitalization or follow-up. The inclusion criteria were as follows: The chief complaint was acute gouty arthritis, and the first visit records were selected for the patients hospitalized for multiple times. The exclusion criteria are as follows:(a) patients who are at risk of death from a critical illness, and (b) those whose examination is incomplete. Data collected from the enrolled neonates included the general status of gout patients (age, sex), previous history (whether they had kidney stones, hypertension, diabetes), blood routine, liver function, kidney function, electrolytes, and lipid profile.

### Data preprocessing and statistical analysis

All data processing and statistical analyses were conducted using the DecisionLinnc platform (DecisionLinnc Core Team, 2023; Hangzhou, China (16), Configure the environment to Python3.10.6). Categorical variables were summarized as frequencies and percentages, and continuous variables were expressed as mean ± standard deviation (SD), and group comparisons were conducted using the Kruskal–Wallis or Mann–Whitney U tests, depending on distribution normality. K-nearest neighbors (KNN) imputation was used to handle below 25% missing laboratory data (e.g., serum creatinine, LDL cholesterol), as these biomarkers often correlate with other variables (e.g., age, BMI). For each missing value, the algorithm imputed estimates based on the k most similar patients (neighbors) using Euclidean distance.

### Class imbalance handling

To address class imbalance in the dataset, we applied Synthetic Minority Oversampling Technique (SMOTE) combined with random undersampling of the majority class. This hybrid approach generated synthetic minority-class samples in the feature space while reducing majority-class instances to achieve a 1:1 class ratio, thereby improving model sensitivity without compromising data integrity. The resampling was exclusively performed on the training set (prior to variable selection) to prevent information leakage into validation cohorts.

### Feature selection

**Figure 1:**
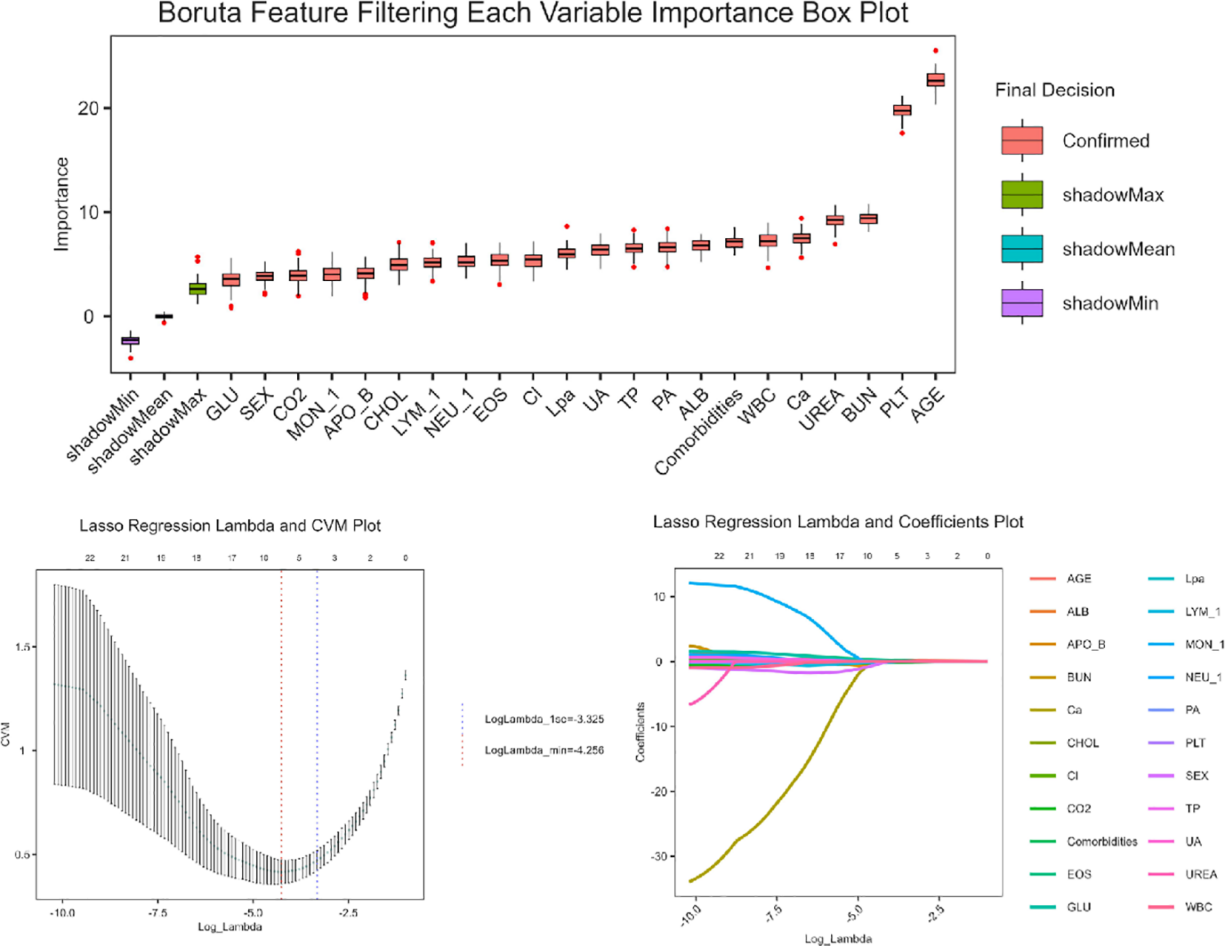
Feature selection using Boruta feature and lasso regression.

Feature selection was conducted in two sequential steps (**Figure 1**): First, Boruta Algorithm, a random forest-based wrapper method was applied to identify the most relevant predictors. This method leverages shadow features and recursive elimination to capture all potential predictive features while minimizing overfitting. Subsequently, least absolute shrinkage and selection operator (LASSO) regression with 10-fold cross-validation was applied to identify the eight most influential predictors, optimizing the penalty parameter (λ) to minimize classification error while maintaining model parsimony. These selected variables were incorporated into a multivariable logistic regression model to generate predictive probabilities. The final model was visualized as a clinically interpretable nomogram.

### Model selection rationale

Each model was trained using the training cohort and evaluated on the independent testing cohort. In this study, five machine learning algorithms were implemented for predictive modeling, each configured with specific hyperparameters as follows (17):

#### Decision tree

A decision tree classifier was constructed using the Gini impurity criterion as the splitting metric. The model was trained with a fixed random state of 1. The splitting strategy was set to “best”, with a maximum depth of 3. The minimum number of samples required to split an internal node was set to 2, and the minimum number of samples required to be at a leaf node was set to 1. The maximum number of features considered during a split was limited to 100, and the maximum number of leaf nodes was also set to 100.

#### Light gradient boosting machine

LGBM was implemented with the GBDT (Gradient Boosting Decision Tree) booster type and a fixed random seed of 1. The learning rate was set to 0.1 to control the contribution of each tree in the ensemble.

#### K-nearest neighbors classifier

The k-nearest neighbors model used the uniform weighting scheme, meaning each of the k neighbors contributes equally to the classification. The number of nearest neighbors (k) was set to 5, and the algorithm type was set to “auto”, which automatically selects the most appropriate algorithm based on the input data.

#### CatBoost

CatBoost, a gradient boosting algorithm optimized for categorical features, was applied with 100 boosting iterations and a tree depth of 10. The learning rate was set to 0.1. The evaluation metric used during training was Logloss, and the random seed was fixed at 1.

#### Gradient boosting decision tree

The GBDT model was trained with a log loss function and a learning rate of 0.1. The number of boosting stages was set to 100. The model used a subsample rate of 1.0, indicating that all training samples were used in each boosting iteration. The splitting quality was evaluated using the Friedman MSE criterion. The minimum number of samples required to split an internal node was set to 2, and the minimum number of samples required at a leaf node was set to 1. The maximum tree depth was 200, with both the maximum number of features and the maximum number of leaf nodes set to 100. A fixed random state of 1 was used for reproducibility.

### Model interpretation

Clarified the Model Used: We now explicitly state that the SHAP values were computed using the final trained model, which was evaluated on the test (or external validation) set. SHapley Additive exPlanations (SHAP) values were used to quantify and visualize each variable’s contribution to model output. SHAP summary plots, force plots, and dependence plots were used to visualize how each feature affected the model’s prediction. This interpretability allows for both clinician understanding and potential clinical decision-making.

### Model evaluation and bootstrap validation

To assess the performance and generalizability of the machine learning models, a bootstrap resampling procedure was conducted. Specifically, the bootstrap process was repeated for 10 iterations, with 80% of the original dataset randomly sampled with replacement in each iteration to form the training set, while the remaining 20% was used for testing. A fixed random seed of 1 was applied to ensure reproducibility.

Performance evaluation was conducted using multiple classification metrics, including accuracy, precision, recall, F1-score, and the area under the receiver operating characteristic curve (AUC). These metrics were calculated for each iteration and averaged to obtain a robust estimate of model performance. Model validation was primarily based on the best-performing algorithm—CatBoost—and bootstrap-based error estimates were used to evaluate the stability and robustness of the predictive outcomes across resampling iterations.

To perform the SE data task, the dataset was randomly split into training and test sets with a training ratio of 0.7, using a fixed random seed of 123 to ensure reproducibility. For model training, the CatBoost algorithm was applied with manual hyperparameter tuning. The number of computational threads was set to 4. Model evaluation was conducted using 10-fold cross-validation (CV) to ensure robust performance estimation. The evaluation metrics included accuracy (ACC), Cohen’s Kappa coefficient (Kappa), and the area under the receiver operating characteristic curve (AUC). In addition, a portion of the data was used for SE prediction.

The research was supported by the Key Research Project of the China Academy of Chinese Medical Sciences(CI2021A01514). All authors have full access to all data in the study and accept responsibility for submitting it for publication.

## Results

### General characteristics

This study included a total of 686 patients, among whom 263 experienced cardiovascular events (CVE group) and 423 did not (non-CVE group). The mean age of the overall cohort was 57.56 years, with CVE patients significantly older than non-CVE patients (67.1 ± 13.7 *vs*. 51.63 ± 14.53 years, P < 0.01, SMD = 1.10). Sex distribution revealed a higher proportion of males in both groups, although the difference was statistically significant (P = 0.03). Comparison of baseline laboratory parameters showed that patients in the CVE group had significantly lower platelet counts (PLT), lymphocyte percentages (LYM), total protein (TP), albumin (ALB), prealbumin (PA), and calcium (Ca), and higher blood urea (UREA), blood urea nitrogen (BUN), creatinine (CREA), uric acid (UA), glucose (GLU), low-density lipoprotein cholesterol (LDL-C), and comorbidity burden (P < 0.05 for all). Additionally, statistically significant differences were observed in CO_2_, sodium (Na), and several lipid-related markers such as LDL-C and GLU levels. Among categorical variables, comorbidity profiles were markedly different between groups: a higher proportion of CVE patients had fewer comorbidities, particularly those without hypertension or diabetes conditions (P < 0.01, SMD = 0.75). Detailed comparisons are presented in **Table 1**.

**Table 1:** Baseline characteristics of patients in the CVE and non-CVE cohort.

Variable names	Level	Overall	0	1	P
n		686	423	263	
AGE		**57.56 ± 16.08**	**51.63 ± 14.53**	**67.1 ± 13.7**	**<0.01**
WBC(×10^9^/L)		7.6 ± 2.41	7.72 ± 2.35	7.39 ± 2.51	0.08
PLT(×10^9^/L)		**267.43 ± 87.09**	**277.67 ± 82.68**	**250.97 ± 91.53**	**<0.01**
NEU(%)		64.94 ± 10.29	64.6 ± 9.81	65.48 ± 11	0.28
LYM(%)		25.85 ± 9.03	26.47 ± 8.88	24.84 ± 9.2	0.02
MON(%)		6.35 ± 1.67	6.3 ± 1.56	6.44 ± 1.84	0.32
EOS(%)		2.22 ± 1.72	2.15 ± 1.59	2.34 ± 1.92	0.16
BAS(%)		0.46 ± 0.27	0.47 ± 0.27	0.45 ± 0.27	0.54
NEU(×10^9^/L)		5.13 ± 2.96	5.11 ± 2.2	5.17 ± 3.9	0.79
LYM(×10^9^/L)		1.9 ± 1.42	1.93 ± 0.61	1.86 ± 2.16	0.55
MON(×10^9^/L)		0.48 ± 0.29	0.48 ± 0.18	0.49 ± 0.42	0.79
E0S(×10^9^/L)		0.16 ± 0.13	0.15 ± 0.11	0.16 ± 0.17	0.27
BAS(×10^9^/L)		0.03 ± 0.02	0.03 ± 0.02	0.03 ± 0.02	0.09
ESR(mm/h)		26.04 ± 23.69	25.43 ± 22.44	27.03 ± 25.59	0.39
TP(g/L)		**70.84 ± 5.51**	**71.58 ± 5.36**	**69.65 ± 5.56**	**<0.01**
ALB(g/L)		**41.2 ± 4.17**	**41.76 ± 3.97**	**40.31 ± 4.33**	**<0.01**
G(g/L)		29.78 ± 4.49	29.97 ± 4.22	29.47 ± 4.9	0.16
A/G		1.43 ± 0.37	1.42 ± 0.24	1.43 ± 0.52	0.85
PA(mg/L)		261.87 ± 63.09	266.53 ± 61.24	254.38 ± 65.39	0.01
FFA(mmol/L)		0.77 ± 3.39	0.89 ± 4.32	0.57 ± 0.16	0.24
UREA(mmol/L)		**5.37 ± 2.28**	**5 ± 1.65**	**5.97 ± 2.94**	**<0.01**
BUN(mmol/L)		**15.01 ± 6.24**	**14.02 ± 4.6**	**16.6 ± 7.97**	**<0.01**
CREA(μmol/L)		**92.93 ± 28.41**	**90.53 ± 22.08**	**96.81 ± 36.05**	**<0.01**
UA(μmol/L)		**476.89 ± 111.14**	**486.8 ± 111.76**	**460.94 ± 108.45**	**<0.01**
K(mmol/L)		4.19 ± 0.36	4.19 ± 0.34	4.2 ± 0.39	0.59
Na(mmol/L)		140.92 ± 2.24	141 ± 2.08	140.79 ± 2.46	0.22
Cl(mmol/L)		102.79 ± 3.19	102.63 ± 3.06	103.05 ± 3.37	0.09
CO2(mmol/L)		**25.11 ± 2.47**	**25.35 ± 2.28**	**24.72 ± 2.72**	**<0.01**
Ca(mmol/L)		**2.32 ± 0.12**	**2.34 ± 0.12**	**2.29 ± 0.1**	**<0.01**
GLU(mmol/L)		**5.9 ± 1.54**	**5.75 ± 1.44**	**6.14 ± 1.66**	**<0.01**
LDL-C(mmol/L)		2.95 ± 0.79	3.02 ± 0.79	2.85 ± 0.79	0.01
VLDL(mmol/L)		0.7 ± 0.52	0.68 ± 0.45	0.72 ± 0.62	0.25
APO-A1(g/L)		1.11 ± 0.19	1.11 ± 0.19	1.12 ± 0.2	0.35
APO-B(g/L)		0.99 ± 0.24	1.01 ± 0.25	0.97 ± 0.23	0.08
Lpa(mg/L)		220.51 ± 351.16	225.16 ± 421.65	213.04 ± 189.61	0.66
CHOL(mmol/L)		4.74 ± 0.84	4.8 ± 0.83	4.64 ± 0.85	0.01
SEX (%)	Female	48 (7.00)	22 (5.20)	26 (9.89)	0.03
	Male	638 (93.00)	401 (94.80)	237 (90.11)	
Kidney stone (%)	No	565 (82.36)	350 (82.74)	215 (81.75)	0.82
	Yes	121 (17.64)	73 (17.26)	48 (18.25)	
Comorbidities (%)	**1**	**321 (46.79)**	**254 (60.05)**	**67 (25.48)**	**<0.01**
	2	76 (11.08)	32 (7.57)	44 (16.73)	
	3	29 (4.23)	14 (3.31)	15 (5.70)	
	4	260 (37.90)	123 (29.08)	137 (52.09)	

WBC, White Blood Cell count; PLT, Platelet count; NEU, Neutrophil count; LYM, Lymphocyte count; MON, Monocyte count; EOS, Eosinophil count; BAS, Basophil count; NEUp, Neutrophil percentage; LYMp, Lymphocyte percentage; MONp, Monocyte percentage; E0Sp, Eosinophil percentage; BASp, Basophil percentage; ESR, Erythrocyte Sedimentation Rate; TP, Total Protein; ALB, Albumin; Gv, Globulin value; AG, Albumin/Globulin Ratio; PA, Prealbumin; FFA, Free Fatty Acids; UREA, Urea concentration; BUN, Blood Urea Nitrogen; CREA, Creatinine; UA, Uric Acid; Kv, Potassium value; Na, Sodium; Cl, Chloride; CO2, Carbon Dioxide content; Ca, Calcium; GLU, Glucose; LDLC, Low-Density Lipoprotein Cholesterol; VLDL, Very Low-Density Lipoprotein; APOA1, Apolipoprotein A1; APOB, Apolipoprotein B; Lpa, Lipoprotein (a); Sex, Sex distribution; n (%), com, Presence of comorbidity; n (%), Group 1, Participants without hypertension or diabetes; Group 2, Participants with both hypertension and diabetes; Group 3, Participants with diabetes only; Group 4, Participants with hypertension only; Kidney, Kidney-related condition, n (%). A P value of less than 0.01 was considered statistically significant, suggesting a strong association between the baseline characteristic and the clinical outcome.

### Predictor screening

**Figure 2:**
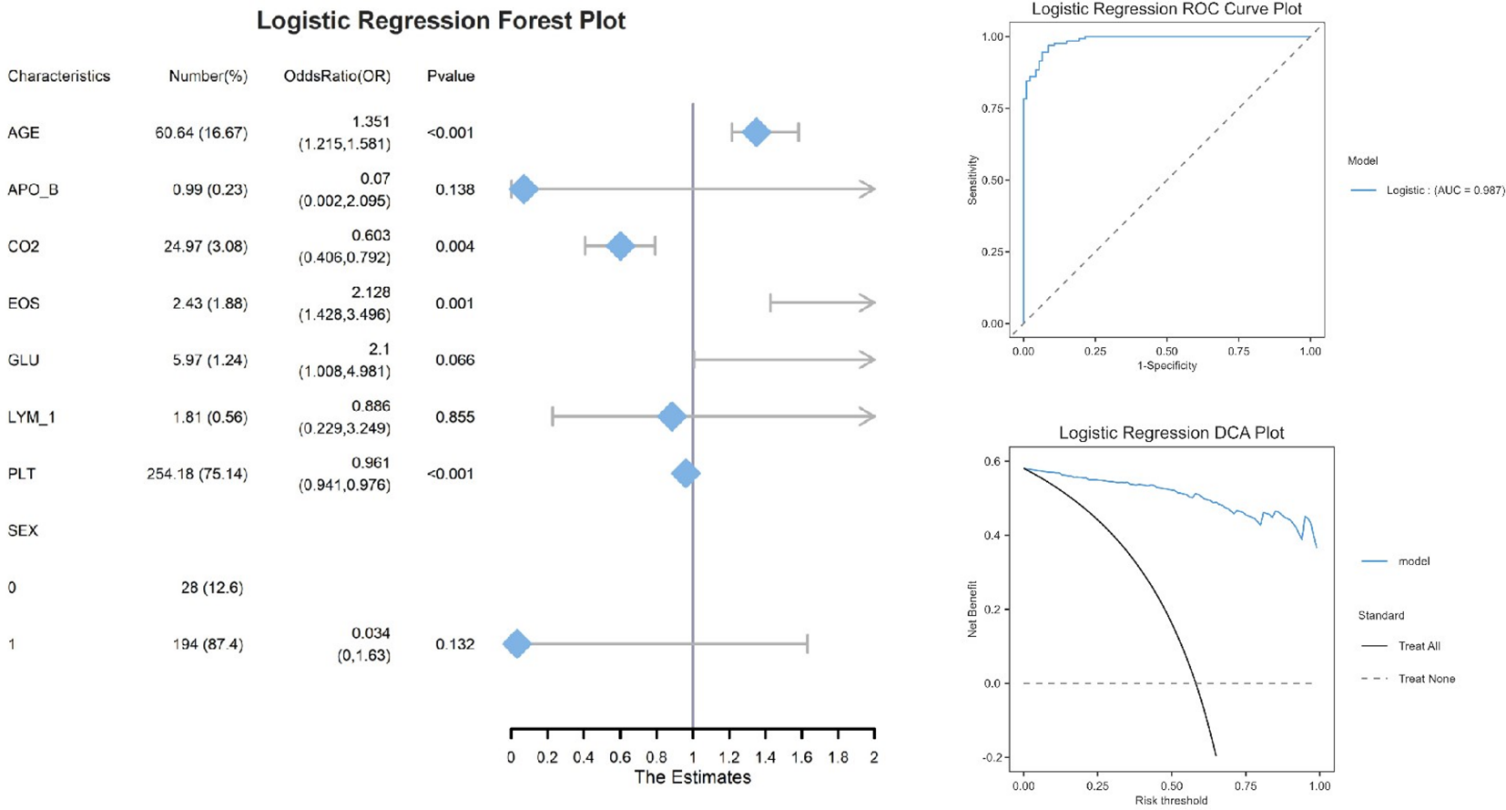
Logistic regression performance metrics.

**Figure 2** displays the distribution of variable importance scores across multiple iterations, comparing original features with synthetic shadow features (random noise variables).Representative features such as age, PLT, BUN demonstrated stable importance across iterations (narrow boxplot ranges), suggesting their strong association with the outcome. Four variables (not shown in figure) fell below the shadowMin threshold and were rejected as irrelevant. The Lasso regression analysis identified a parsimonious set of 8 clinically relevant predictors from the initial 39 variables (**Figure 2**): sex, age, PLT, EOS, LYM, CO2, GLU, APO-B. APO-B (β=−0.02, negative association), suggesting a potential protective role in the disease progression, EOS (β=+0.22) and GLU (β=+0.06), indicating their positive correlations with adverse outcomes.

**Figure 3:**
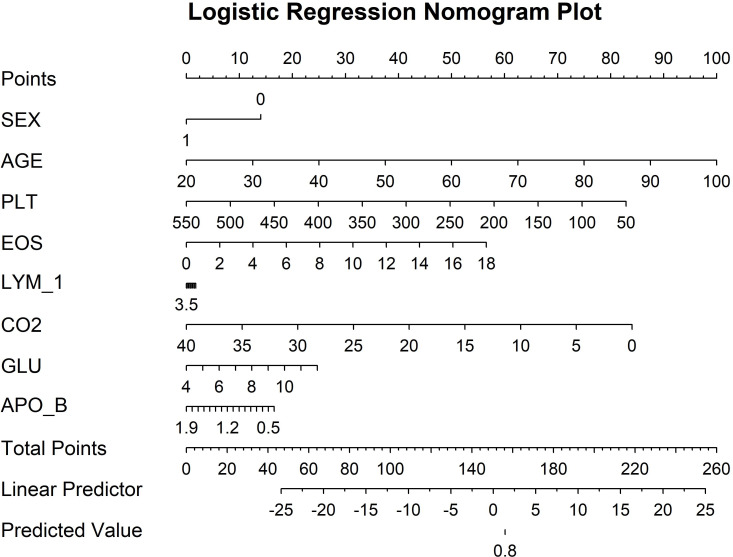
Logistic regression nomogram plot.

The logistic regression analysis (**Figure 2**) identified four significant predictors of [outcome]: AGE (OR=1.351, 95% CI:1.215–1.581, P<0.001), CO2 (OR=0.603, 95% CI:0.406–0.792, P=0.004), EOS (OR=2.128, 95% CI:1.428–3.496, P=0.001), and PLT (OR=0.961, 95% CI:0.941–0.976, P<0.001). Notably, AGE and EOS exhibited strong positive associations, while higher CO2 and PLT levels were protective. Variables such as APO_B (P=0.138) and SEX (P=0.132) did not reach statistical significance, possibly due to limited effect sizes or sample heterogeneity. The logistic regression model demonstrated excellent discriminative ability, with an area under the ROC curve (AUC) of 0.987. Decision curve analysis demonstrated that the logistic regression model provided superior net benefit compared to the ‘Treat All’ or ‘Treat None’ strategies across a clinically relevant risk threshold range (0.2–0.6). For example, at a threshold probability of 30% (a common cutoff for clinical intervention), the model yielded a net benefit of 0.45, whereas ‘Treat All’ and ‘Treat None’ resulted in 0.25 and 0, respectively. This suggests that using the model to guide decisions could prevent unnecessary treatments for 20% of patients without missing high-risk cases. The nomogram (**Figure 3**) integrated eight clinically accessible variables, with AGE and EOS contributing the highest point weights.

**Figure 4:**
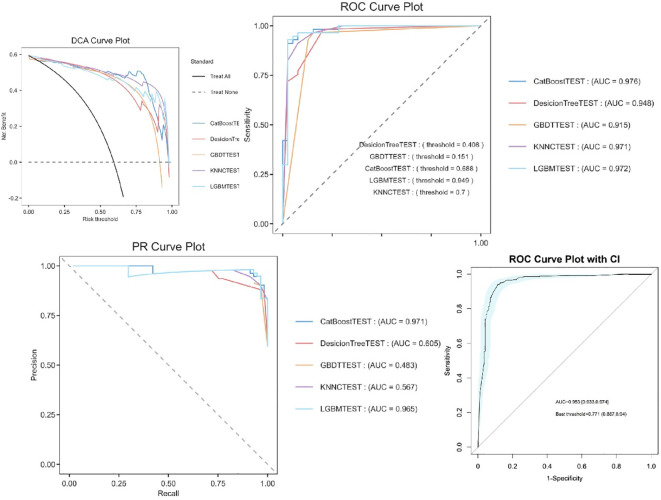
Machine learning model performance.

The experimental results demonstrate that CatBoost exhibited superior classification performance, achieving near-perfect AUC values of 0.976 (**Figure 4**). The 95% confidence intervals for CatBoost (AUC range: 0.940-0.972) showed narrow bands without overlap with other models, indicating statistically significant superiority and high prediction stability. PR curve analysis further validated the exceptional performance of CatBoost (AUC=0.971) and RF (AUC=0.969) in handling potential class imbalance, while decision trees showed markedly inferior performance (AUC=0.539). Calibration curve assessment revealed that CatBoost produced the most accurate probability estimates, with predictions closely aligned to the ideal diagonal, suggesting its probability outputs can be reliably interpreted as confidence measures.

### Machine learning model performance

Comparative analysis of five machine learning models revealed that ensemble methods consistently outperformed single-model approaches (**Supplementary 8**). The CatBoost learner achieved the highest discriminative ability (AUC=0.953, 95% CI: 0.933–0.974), with a sensitivity of 88.7% and specificity of 94% at the optimal threshold (0.771). LightGBM (AUC=0.972) and KNNC (AUC=0.971) followed closely, while Decision Trees (AUC=0.948) and GBDT (AUC=0.915) exhibited limited performance, likely due to their inability to handle complex feature interactions.

### SHAP-based model interpretability analysis

**Figure 5:**
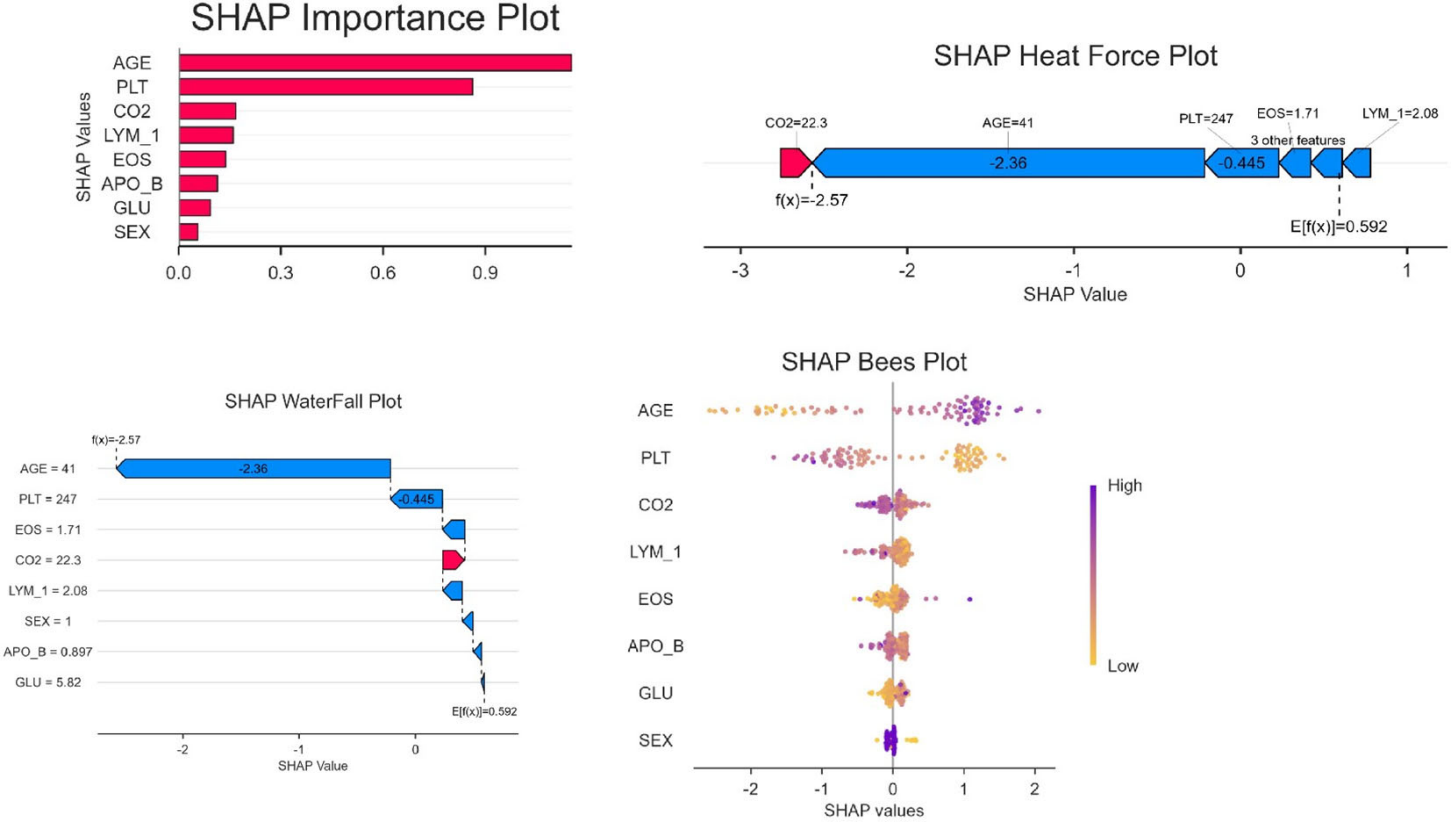
SHAP model interpretation.

The SHAP feature importance analysis (**Figure 5**) identified AGE as the most influential predictor (mean |SHAP value|=0.9), followed by PLT (0.6) and CO2 (0.45). In contrast, demographic variables such as SEX (0.1) showed minimal contributions, suggesting that clinical biomarkers drive the model’s decisions more strongly than baseline characteristics. The top SHAP features (AGE, PLT) correspond to the variables retained in the logistic regression nomogram (**Figure 2**), reinforcing their biological plausibility. CatBoost’s superior AUC may stem from its ability to capture non-linear relationships in high-importance features like AGE, whereas simpler models (e.g., Decision Trees) underutilized these patterns.

### Model evaluation and bootstrap validation

**Figure 6:**
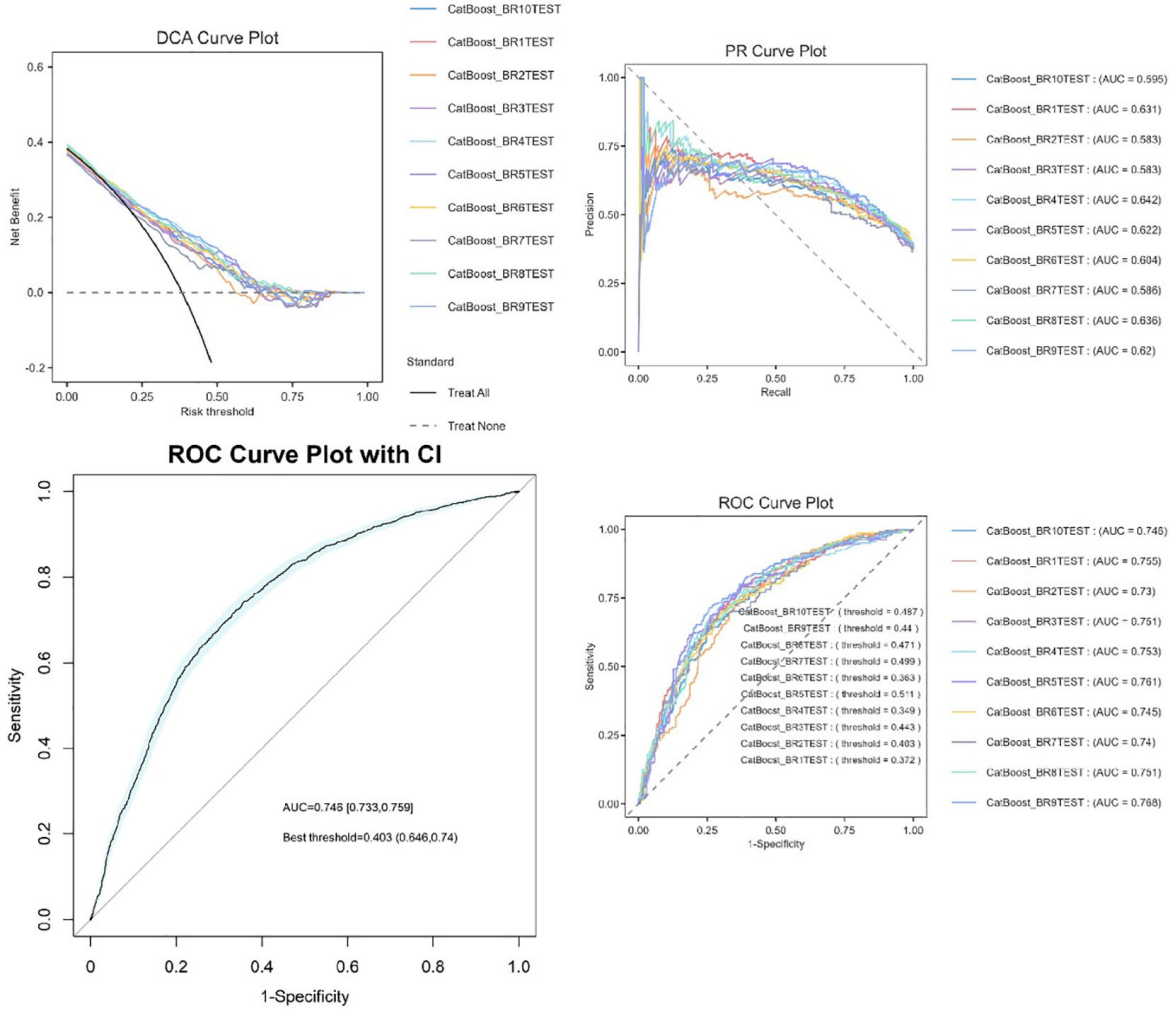
Bootstrap-validated ROC curves of CatBoost models.

Ten bootstrap-validated CatBoost models (BR1TEST-BR11TEST) demonstrated moderate to strong discriminative ability, with AUC values ranging from 0.686 to 0.744 (median AUC=0.726, IQR: 0.711–0.731). Classification thresholds varied between 0.318 and 0.476, reflecting dataset heterogeneity (**Figure 6**). The most stable model (BR7TEST) achieved the highest AUC (0.744) at a threshold of 0.447. Threshold variability (0.318–0.476) underscores the importance of tailoring decision cutoffs to clinical priorities—selecting BR7TEST (high specificity) for confirmatory testing or BR6TEST (low threshold=0.318) for sensitive screening.

## Discussion

Gout, recognized as the most prevalent inflammatory arthritis worldwide, is pathologically rooted in sustained hyperuricemia (18). Epidemiological investigations demonstrate a 20% elevation in gout incidence per 1 mg/dL increment in serum urate levels, concurrently exhibiting significant comorbidity with metabolic syndrome components including hypertension, diabetes mellitus, and dyslipidemia (19, 20). Despite substantial advancements in contemporary medicine, clinical management of gout persists as a formidable challenge: over 60% of patients fail to achieve target serum urate control (<6 mg/dL), resulting in progressive disease burden (4). Notably, the mechanistic interplay between gout and CVDs warrants in-depth elucidation. Large-scale cohort studies reveal that gout patients experience 28% higher all-cause mortality (aHR=1.28, 95%CI 1.15-1.42) compared to the general population, with cardiovascular-related mortality showing a more pronounced 38% increase (aHR=1.38, 95%CI 1.21-1.58) (21). However, clinical practice data indicate only 25% of acute gout patients undergo systematic cardiovascular risk assessment within one month post-attack, underscoring substantial optimization potential in current therapeutic strategies (22).

These findings align with existing evidence linking metabolic syndrome to cardiovascular risk: Age-related risk accumulation: Each decade beyond 65 years confers exponential CVDs risk elevation (HR=1.62, 95%CI 1.38-1.91), predominantly driven by accelerated vascular remodeling associated with 12.7% annual arterial stiffness progression (23, 24). Glycometabolic dysregulation synergy: In gout patients with diabetes, each 1 mmol/L fasting glucose increment correlates with)0.34 ng/L elevation in high-sensitivity cardiac troponin T (hs-cTnT) (β=0.34, p=0.003) (25). Mechanistically, sustained hyperglycemia (>7.8 mmol/L) activates PKC-β/NADPH oxidase pathways, inducing 2.1-fold ROS overproduction and elevating endothelial apoptosis to 38.5%; Insulin resistance multimodality effects (26): Hyperinsulinemia (fasting insulin ≥15 μIU/mL) reduces endothelial nitric oxide synthase (eNOS) activity by 57% while enhancing vascular smooth muscle cell calcium influx by 83% ([Ca^2+^]i=421 ± 25 nM *vs*. control 228 ± 18 nM) via PI3K/Akt/mTOR signaling, culminating in medial wall thickening (IMT=1.12 ± 0.11 mm *vs*. 0.89 ± 0.09 mm (27, 28); Elevated blood pressure will lead to the inhibition of reactive oxygen species and nitric oxide production, damage to endothelial cells, and lead to the development of atherosclerosis. VLDL and abdominal residual particles accumulate together in the dysfunctional subendothelial vascular wall. Oxidative stress induces oxidative modification of LDL particles and accumulation of oxidized LDL in macrophages, leading to pro-inflammatory macrophage response, excessive macrophage apoptosis and endothelial cell activation, leading to persistent vascular inflammation in atherosclerotic lesions (29–32). Notably, calcium dysregulation emerges as a pivotal mechanism in gout-CVDs comorbidity. Basic research confirms urate crystals induce ATP over-release (+142%, p<0.01) via LRRC8 channel activation (33), triggering intracellular calcium overload ([Ca^2+^]i=512 ± 23 nM *vs*. 289 ± 18 nM control) through P2Y2 receptor signaling (34). This calcium dyshomeostasis promotes atherosclerosis via dual pathways: Inducing mitochondrial membrane potential depolarization (37% ΔΨm reduction) in endothelial cells; Activating calcineurin/NFAT pathways to enhance smooth muscle cell migration (2.3-fold increase) (35). Importantly, febuxostat may elevate arrhythmia risk through RyR2-mediated calcium cycling alterations (28% open probability increase), necessitating enhanced therapeutic monitoring (36).

Platelets play a key role in blood clotting and thrombosis. Hyperuricemia (HUA) has been identified as an independent risk factor for cardiovascular diseases. Elevated uric acid levels may promote platelet activation and aggregation by triggering mechanisms such as oxidative stress, endothelial dysfunction, vascular smooth muscle cell proliferation, and inflammatory responses, thereby increasing the risk of cardiovascular events (37). Studies have shown that urate directly affects immune cell populations by altering cytokine expression, modifying chemotactic responses, promoting differentiation, and inducing immune cell activation through interactions with resident tissue cells (38). HUA may enhance oxidative stress by activating NOD-like receptor protein-3 inflammasome induced inflammation, interfering with cardiac cell energy metabolism, affecting antioxidant defense system, and stimulating the production of reactive oxygen species, ultimately leading to decreased cardiac function (39). In patients with gout, serum albumin levels may be related to the risk and outcomes of cardiovascular events. Even in patients with normal glomerular filtration rates, albuminuria was associated with an increased risk of heart failure.

Cardiovascular Injury Mechanisms Involving Ion Channel Dysregulation and Oxidative Stress Activation, Hypocapnia in gout patients is frequently triggered by impaired renal function or lactic acid accumulation. Its mechanisms of cardiovascular injury primarily involve ion channel dysregulation and activation of oxidative stress. Oxidative Stress Activation: The hypocapnic environment upregulates xanthine oxidase (XO) expression via activation of the NF-κB signaling pathway, increasing XO activity in endothelial cells and elevating superoxide anion production. This accelerates the oxidative modification of low-density lipoprotein (LDL) (40). Oxidized LDL (oxLDL) not only promotes foam cell formation but also activates matrix metalloproteinase-9 (MMP-9), which degrades collagen within the fibrous cap of atherosclerotic plaques. This directly compromises plaque stability and accelerates cardiovascular damage (41). Clinical studies confirm a positive correlation between serum XO activity and the volume of the lipid core within carotid artery plaques in gout patients (42).

From 2013 to 2023, significant changes in managing gout with cardiovascular comorbidities, including updated guidelines and treatment regimens, may profoundly influence the transferability of machine learning models. In gout management, traditional treatments such as non-steroidal anti-inflammatory drugs (NSAIDs), colchicine and glucocorticoids remain dominant. However, there’s a growing use of new uric acid-lowering drugs, such as febuxostat and uricase. Besides, personalized uric acid-lowering goals are now more emphasized, with target levels often set at < 6 mg/dL or even < 5 mg/dL, tailored to individual patient needs. Meanwhile, the cardiovascular field has introduced new anticoagulants (e.g., DOACs), PCSK9 inhibitors, and SGLT2 inhibitors, alongside updated guidelines for hypertension and heart failure, such as stricter blood pressure targets and recommendations for novel therapies. Furthermore, extensive research into the gout-cardiovascular disease link has established hyperuricemia as an independent cardiovascular risk factor, prompting adjustments to risk assessment models like the ACC/AHA risk score. These changes can degrade the predictive performance of models that were trained on earlier data when they are applied to contemporary cohorts, as the underlying feature distributions, such as medication patterns, serum uric acid levels, and cardiovascular risk factors, have shifted significantly. Consequently, before deployment, each model must be rigorously evaluated for accuracy drift, generalizability and interpretability in light of these distributional shifts.

In addition, many medications can trigger acute attacks of gout. Diuretics, such as furosemide and hydrochlorothiazide, as well as antihypertensive drugs containing diuretics are common culprits. Recent cases predominantly involve postmenopausal women using diuretics for cardiovascular or kidney diseases. Such patients often present with mild gouty arthritis, rapid nodule formation, and frequent misdiagnosis as osteoarthritis. Aspirin exerts a dual effect on uric acid metabolism. In older adults, even small dose adjustments can precipitate harm, so dose changes in the elderly should be monitored and its use reduced during acute gout attacks.

## Conclusion

Our analysis highlights the hierarchical contributions of key factors to the risk of cardiovascular disease (CVD) in gout patients, with age emerging as the strongest predictor. This aligns with established evidence that aging accelerates arterial stiffness and reduces renal urate excretion, synergistically promoting CVD progression. Sex differences further modulate risk, with males exhibiting higher gout-related CVD incidence due to androgen-driven urate overproduction, while postmenopausal females approach similar risk levels following estrogen decline. Among novel biomarkers, elevated eosinophil counts (EOS) may reflect IL-4/IL-13-mediated vascular inflammation, though longitudinal studies are needed to confirm causality. ApoB underscores the role of atherogenic lipoproteins in gout-CVD comorbidity, potentially exacerbated by urate crystal-induced endothelial injury. Conversely, lymphopenia (LYM) suggests impaired immunoregulation in progressive disease. These findings advocate for age- and sex-stratified CVD screening in gout, while positioning EOS and ApoB as potential therapeutic targets. Future research should explore whether eosinophil suppression or lipid-lowering therapies mitigate gout-specific CVD pathways.

## Strengths and limitations

This study innovatively integrates clinical parameters with machine learning to establish the first East Asian-specific gout-CVDs prediction model. Key limitations warrant attention: First, temporal constraints: Cross-sectional design limits causal inference - longitudinal validation (≥5 years) recommended. Second, pharmacological confounders: Unadjusted diuretic effects (OR=1.89) and aspirin’s dose-dependent impacts. Finally, phenotypic heterogeneity: Undifferentiated gout subtypes (mono- *vs*. polyarticular) may introduce classification bias.

The MCID estimate (5–10% change in predicted risk) and the identified DCA threshold range (0.15–0.30) are derived from the current dataset and may be influenced by the specific patient population and clinical practices from 2013–2023. Although the model performed well in internal cross-validation, its generalizability remains uncertain because it has not yet been validated on external cohorts representing diverse clinical settings. Due to time constraints and the unavailability of suitable external datasets with specific screening for gout and cardiovascular comorbidities, validation was limited to internal k-fold cross-validation. Additionally, the continuous, rapid changes in gout and cardiovascular disease management strategies, diagnostic guidelines, and pharmacotherapy from 2013 to 2023 are likely to produce dataset shift, which in turn may constrain the model’s transferability to contemporary clinical practice. Future investigations should therefore emphasize external validation on multi-center cohorts collected after 2023 to confirm the model’s robustness, generalizability, and real-world applicability. Furthermore, integrating adaptive or transfer learning techniques to mitigate temporal dataset drift will enhance the model’s clinical utility in routine care.

## Data Availability

The raw data supporting the conclusions of this article will be made available by the authors, without undue reservation.
