# Supplementary figures and images for: Establishment and evaluation of a model for clinical feature selection and prediction in gout patients with cardiovascular diseases: a retrospective cohort study
Source: Front Endocrinol (Lausanne). 2025 Oct 10;16:1599028. doi: 10.3389/fendo.2025.1599028 (PMC12549251; doi:10.3389/fendo.2025.1599028)

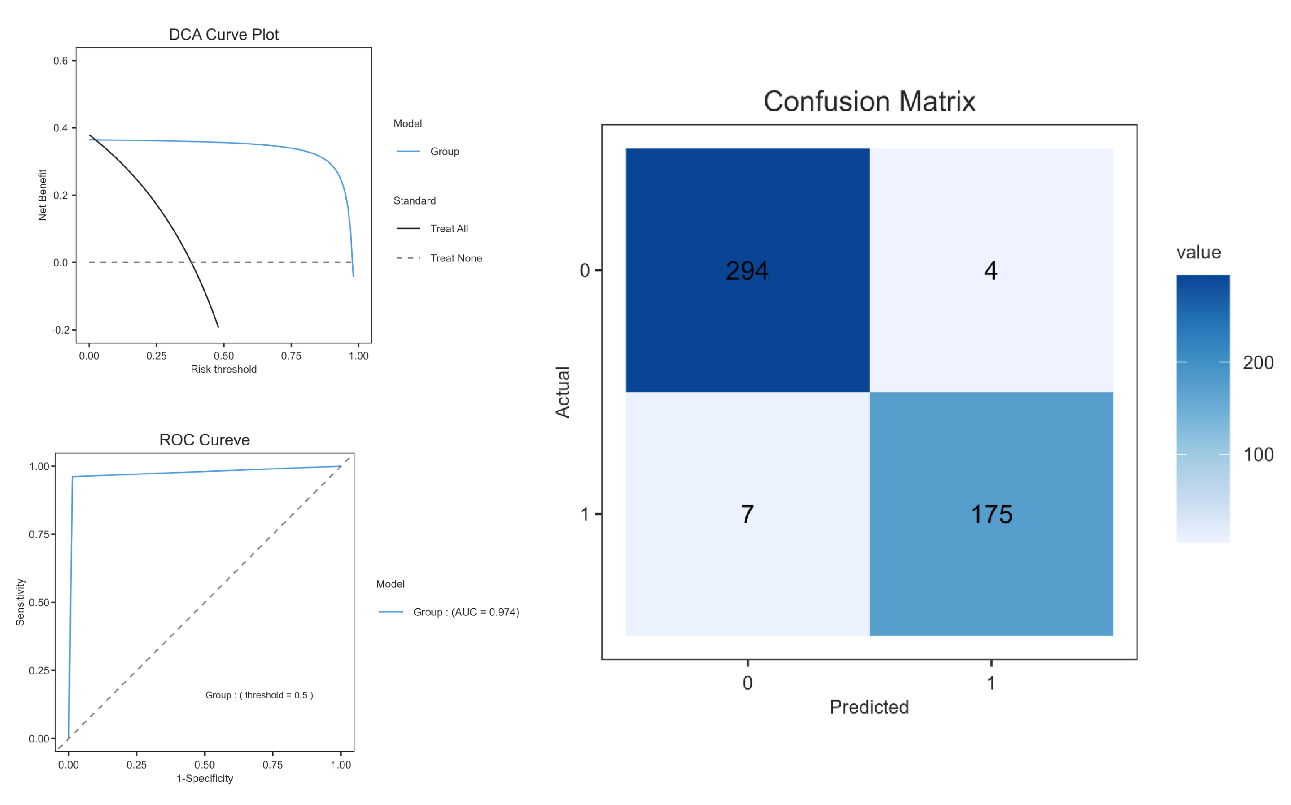

Supplement: Supplementary file 1 [file DataSheet1.zip › Supplements/12-Machine learning confusion matrix.png]

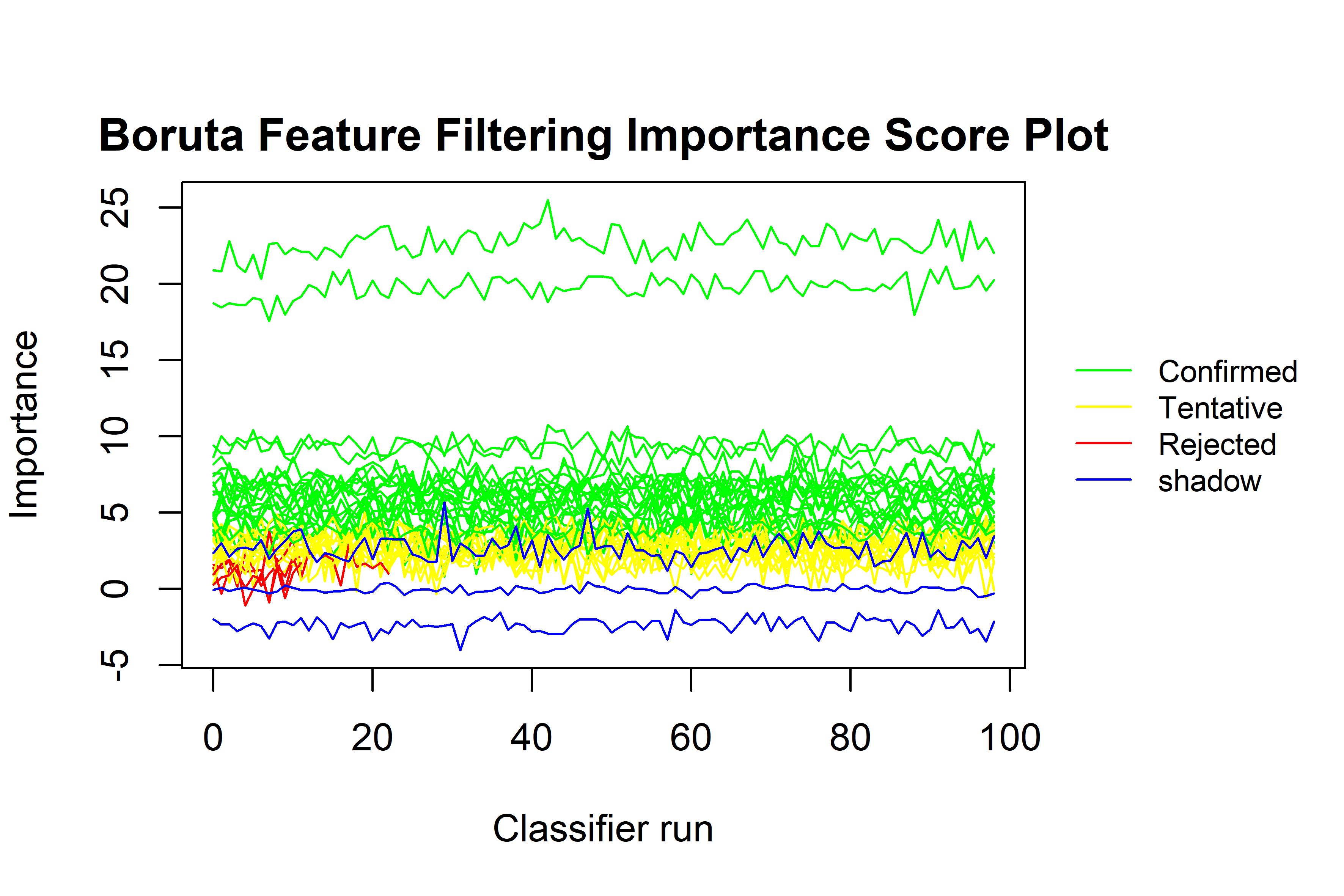

Supplement: Supplementary file 1 [file DataSheet1.zip › Supplements/3-Boruta feature screening importance score chart.jpg]

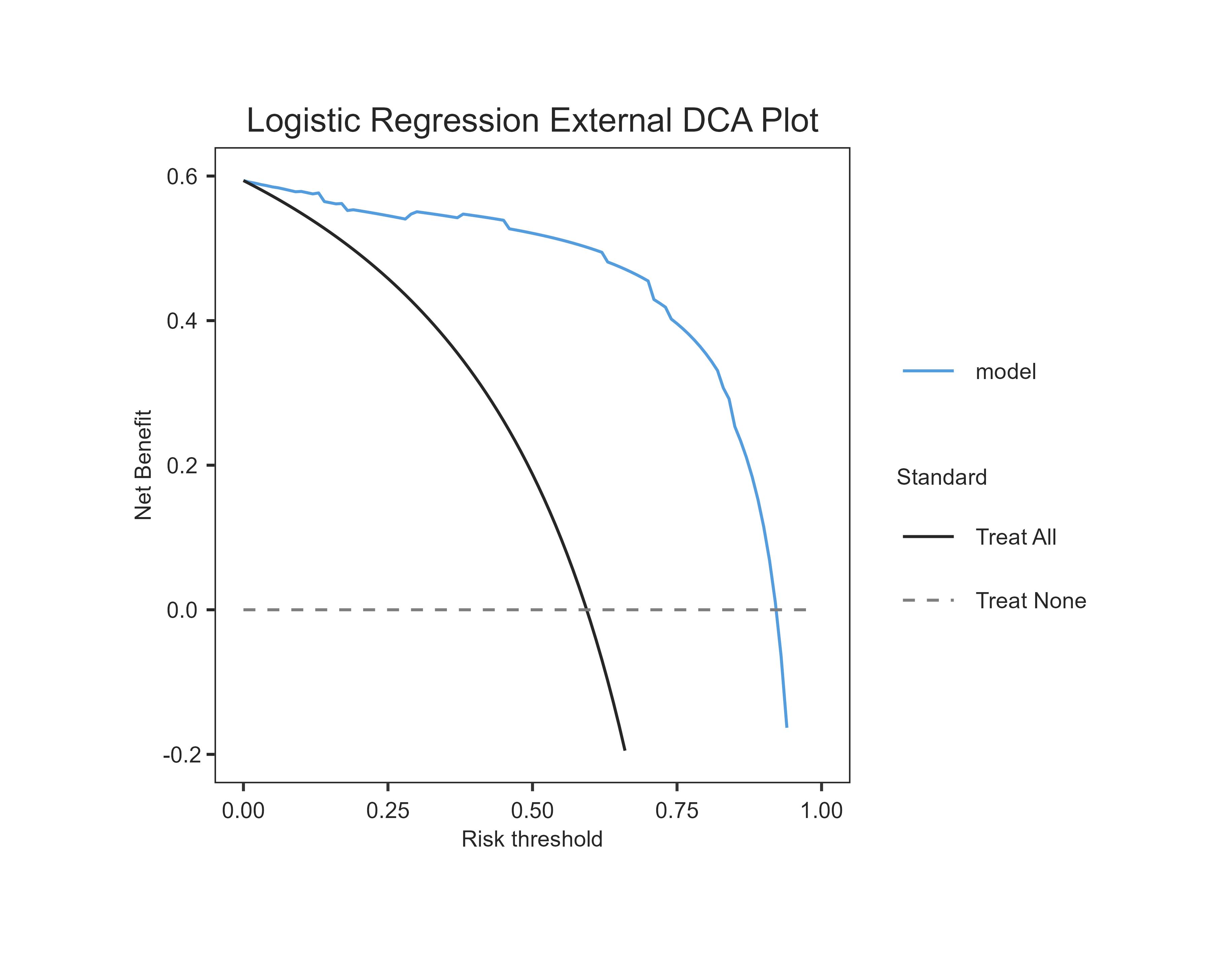

Supplement: Supplementary file 1 [file DataSheet1.zip › Supplements/6-LR External verification DCA.jpg]

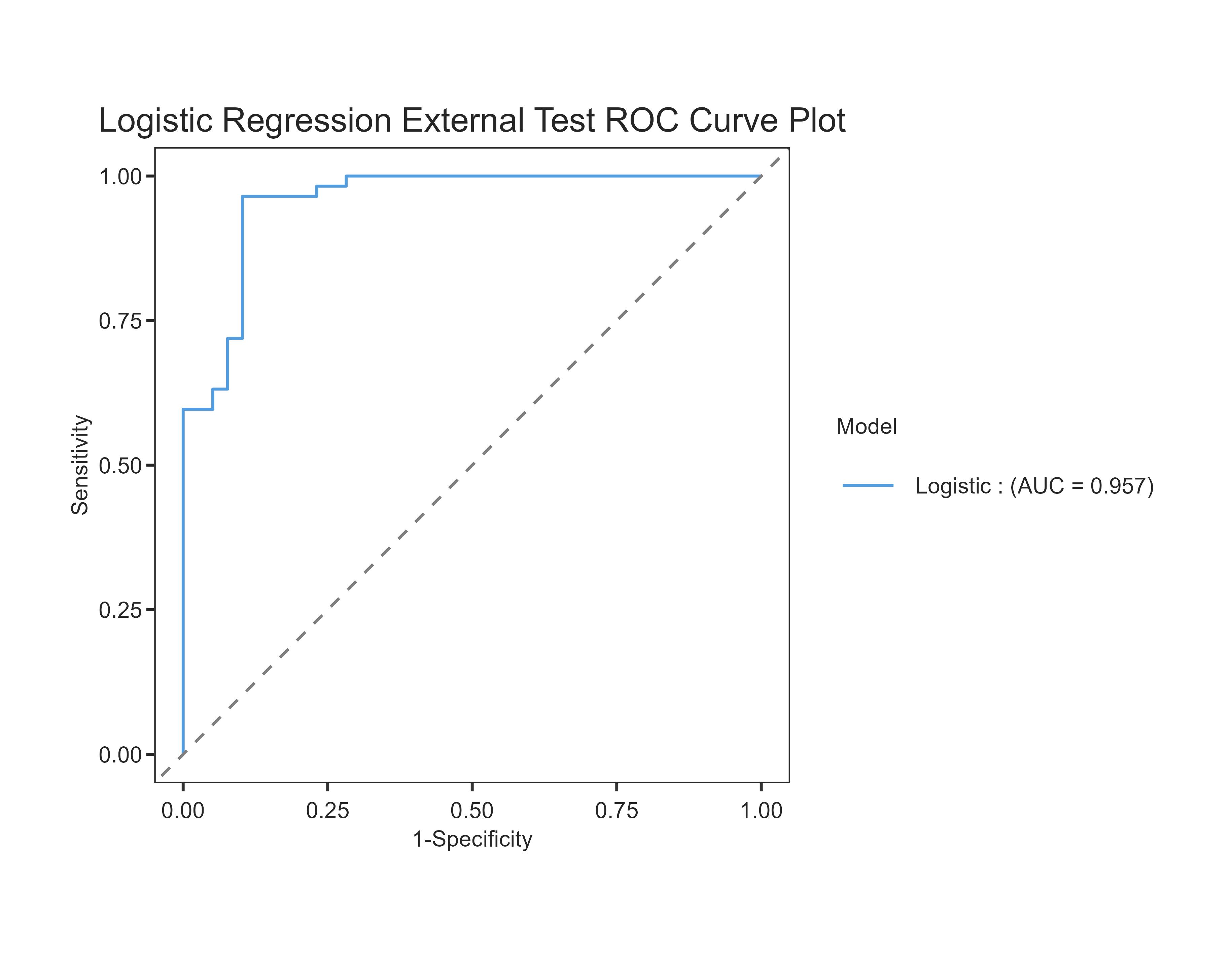

Supplement: Supplementary file 1 [file DataSheet1.zip › Supplements/6-LR External verification ROC.jpg]

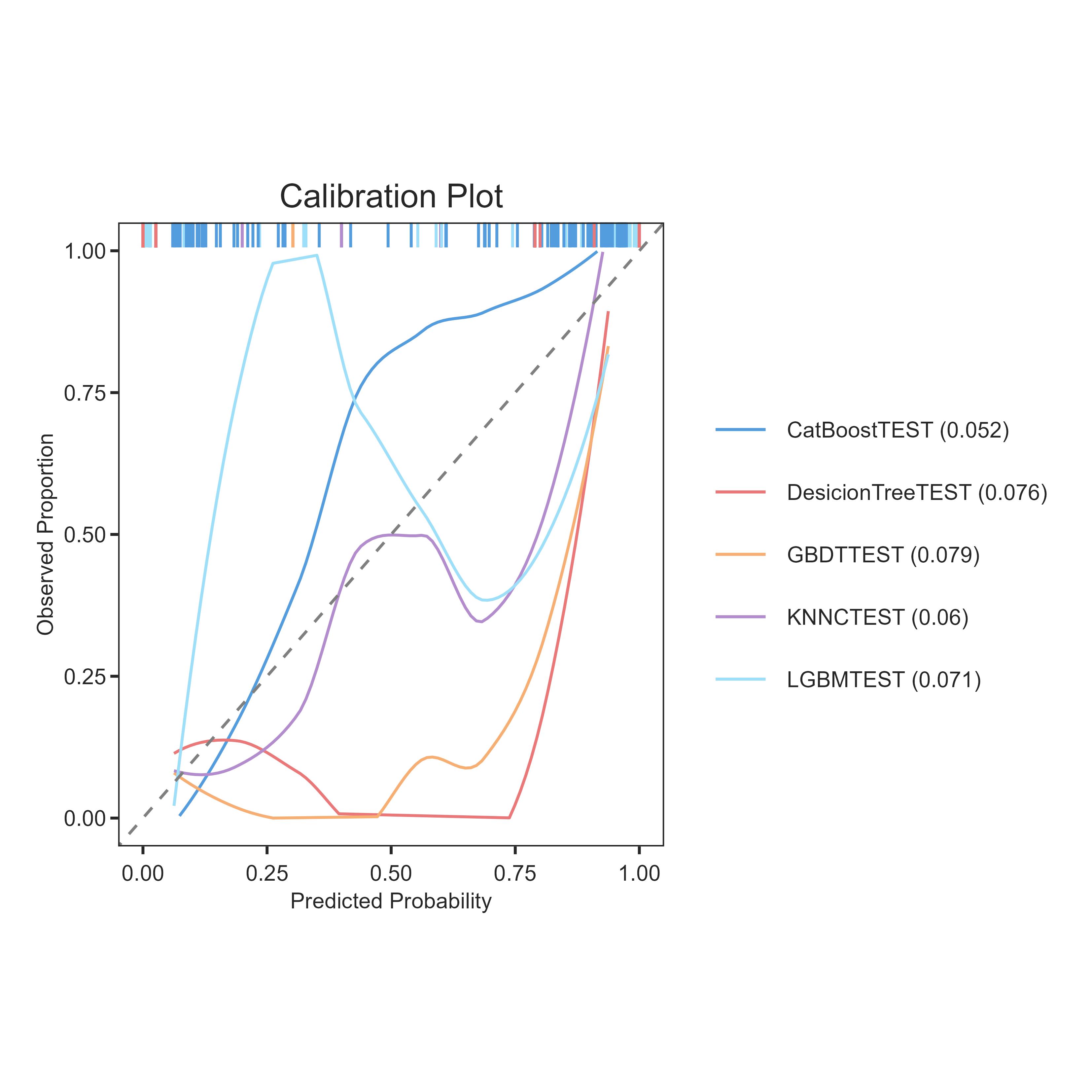

Supplement: Supplementary file 1 [file DataSheet1.zip › Supplements/7-Multi-model evaluation calibration curve.jpg]

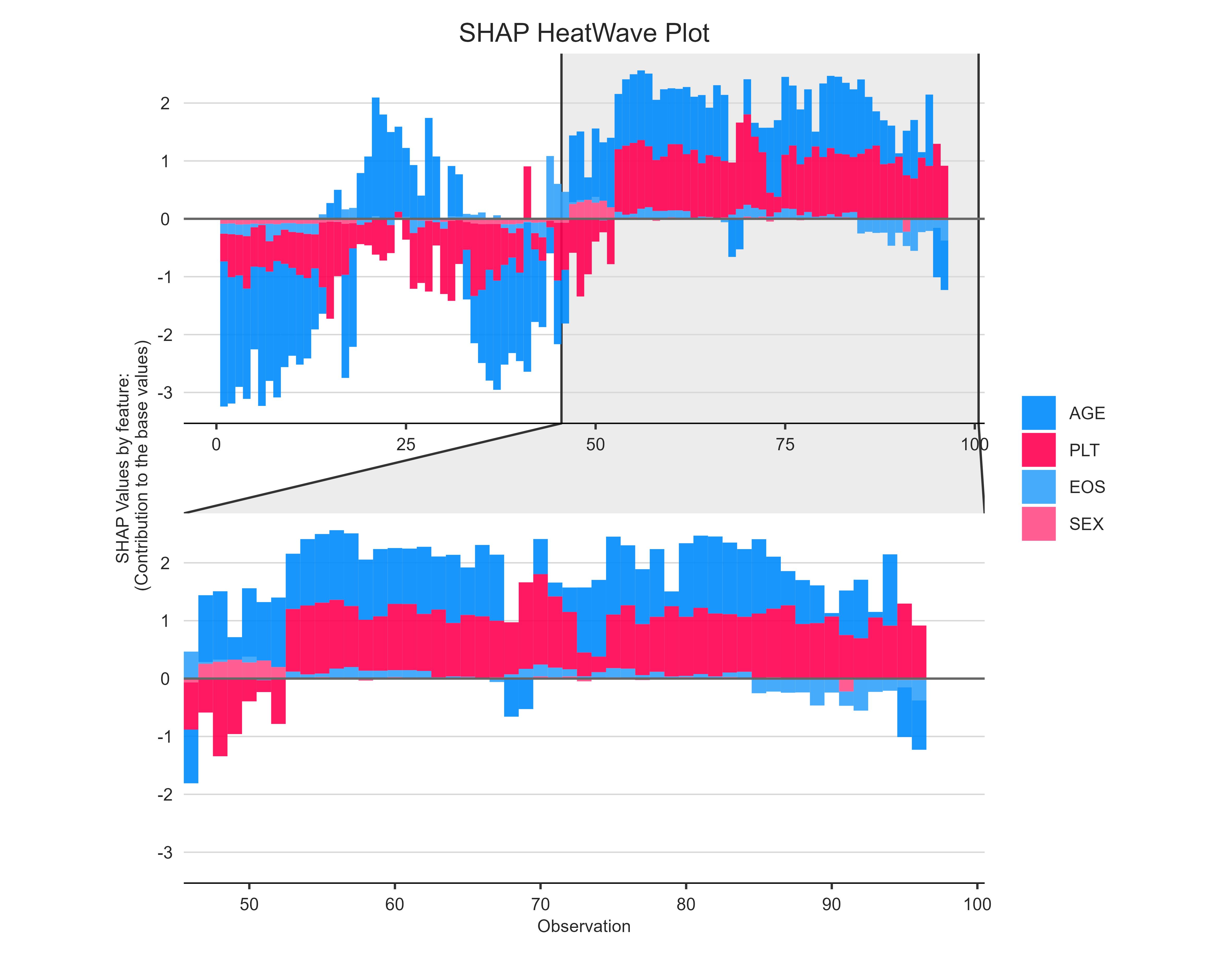

Supplement: Supplementary file 1 [file DataSheet1.zip › Supplements/9-SHAP HeatWave Plot.jpg]

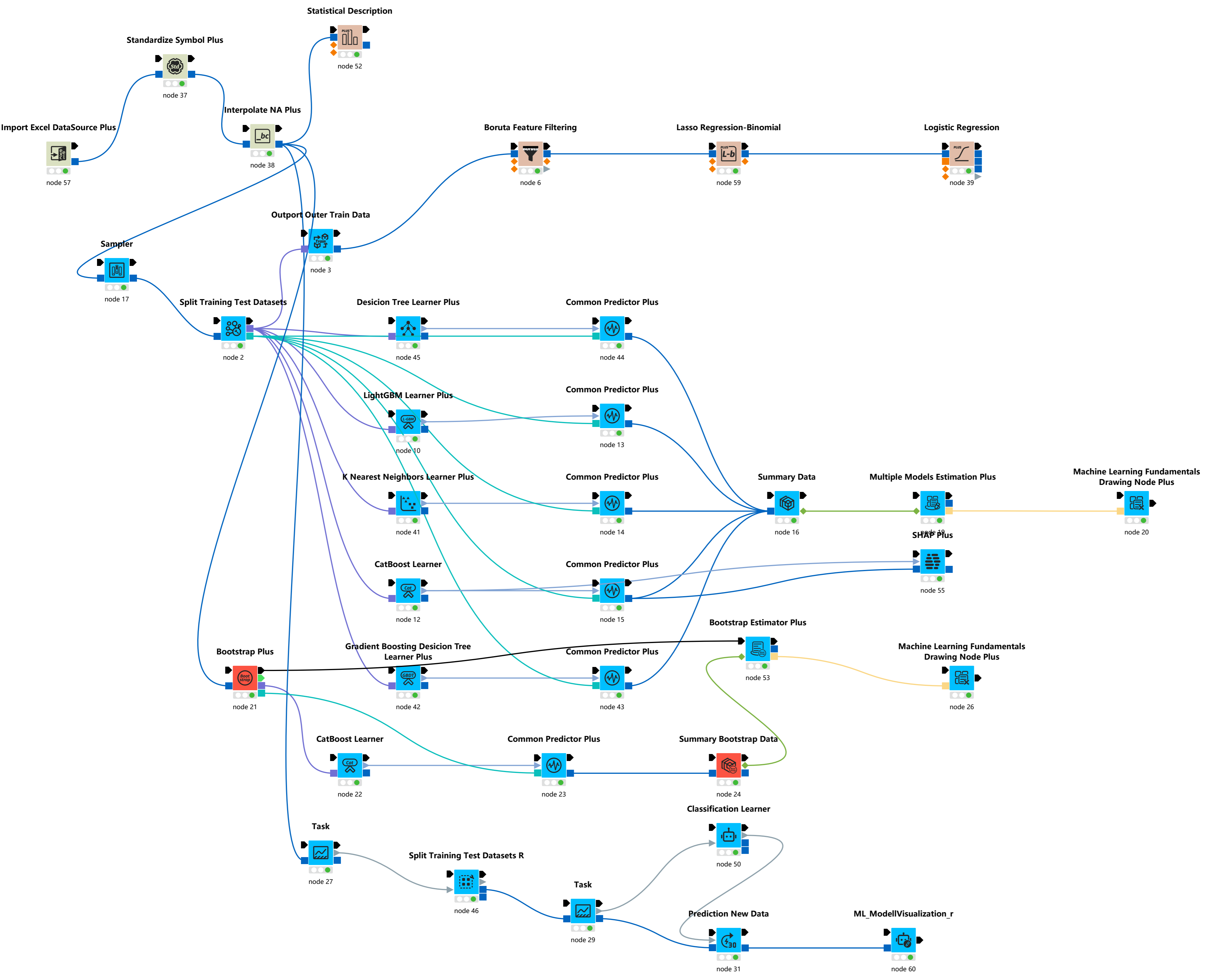

Supplement: Supplementary file 1 [file DataSheet1.zip › Supplements/ML proposal.pdf]
